# Role of anti-Ro autoantibodies in systemic lupus erythematosus patients with recurrent myositis

**DOI:** 10.1186/ar3991

**Published:** 2012-09-27

**Authors:** S Umer, S Hayat, G Caldito, SM Berney

**Affiliations:** 1 Louisiana State University Health Sciences Center, Shreveport, LA, USA

## Background

Anti-SSA (Ro) autoantibodies have been detected in primary Sjögren's syndrome as well as in patients with other systemic autoimmune diseases including systemic lupus erythematosus (SLE). Recent studies have suggested a possible association of anti-Ro antibodies in patients with systemic sclerosis and myositis [1]. These autoantibodies have also been detected in patients with the anti-synthetase antibody syndrome [2]. We investigated the association of anti-Ro antibodies in SLE patients with myositis.

## Methods

In this retrospective study, we reviewed the records of 160 patients with SLE followed in the lupus clinic at LSU Health Sciences Center, Shreveport from 2000 to August 2012.

## Results

We identified 16 patients (10%) with myositis and SLE. Patients with possible secondary cause of myositis, such as thyroid disease and drug, were excluded. All of the patients were females. Fourteen patients (87.5%) were African Americans and two (12.5%) were Caucasians with ages that ranged from 15 to 69 years. Among these 16 patients, 12 (75%) were found to have anti-Ro autoantibodies. The remaining four patients were not tested for anti-Ro. Five patients (41.7%) with higher levels of anti-Ro antibodies at the beginning of their disease experienced recurrences of the myositis. Average anti-Ro was compared between patients with and without recurrences to determine association between myositis recurrence and anti-Ro. A nonparametric test (Wilcoxon rank sum test) was used to do the comparison due the observed non-normal distribution of anti-Ro and the small sample sizes (five and seven) for the two groups compared. Mean ± SD for the recurrence and nonrecurrence groups was 470.4 ± 347.3 and 153.5 ± 113.4, respectively. Using the Wilcoxon rank sum test, the *P *value for the comparison was 0.06. This was not significant at the 5% level but significant at the 7% level (*P *< 0.07). The observed nonsignificance at the 5% level was due to the small sizes (five and seven) for the two groups compared

## Conclusion

The observed results show possible association between myositis recurrence and anti-Ro antibody level. Prospective studies are required to further investigate this association. Myositis in SLE may be under-reported secondary to a lack of awareness of this association, which precludes the simultaneous testing of these autoantibodies along with muscle enzymes. This evaluation is vital during the beginning of disease and with flares because the ongoing muscle damage may be masked and undertreated by the subsequent therapy.
